# Aging, oxidative stress, and cataracts: Therapeutic prospects and translational insights into peroxiredoxin 6

**DOI:** 10.1016/j.preteyeres.2026.101444

**Published:** 2026-01-21

**Authors:** Eri Kubo, Bhavana Chhunchha, Dhirendra P. Singh

**Affiliations:** a Department of Ophthalmology, University of Kanazawa, 1-1 Daigaku, Kahoku-gin, Ishikawa, 920-0293, Japan; b Department of Ophthalmology and Visual Sciences, University of Nebraska Medical Center, Omaha, NE, 68198, USA

**Keywords:** Peroxiredoxin 6, Oxidative stress, Cataractogenesis, aging, Death signaling, Inflammatory response, Selenoproteins

## Abstract

Selenium-independent peroxiredoxin 6 (Prdx6) is a unique member of the peroxiredoxin family, which protects cells from various stressors by regulating reactive oxygen species (ROS) and maintaining survival signaling. As a multifunctional "moonlighting" protein, Prdx6 exhibits glutathione peroxidase (GPx), acidic calcium-independent phospholipase A2, and lysophosphatidylcholine acyltransferase activities, enabling it to reduce ROS. Loss of Prdx6, owing to dysregulation of its transactivator nuclear factor erythroid 2-related factor 2 or aberrant oxidative post-translational modifications from aging or oxidative stress, disrupts cellular homeostasis and triggers inflammatory or non-inflammatory cell death, including apoptosis and pyroptosis. Similar to GPx4, Prdx6 exhibits selenium-independent peroxidase activity and possesses phospholipid hydroperoxide–reducing GPx activity. A novel function of Prdx6 in facilitating selenium utilization was identified recently; that is, it enhances the expression and activity of selenoproteins, especially GPx4, and prevents ferroptosis. Conversely, Prdx6 deficiency reduces selenoprotein levels and promotes ferroptosis. Nevertheless, the molecular mechanisms through which Prdx6 modulates cell death and survival, particularly under aging and oxidative stress conditions contributing to cataractogenesis, remain unclear. In this review, we summarize the current knowledge of Prdx6 regulation and activity during oxidative stress and aging, highlighting its role in inflammatory and non-inflammatory signaling that contributes to eye lens pathology and cataract formation. Additionally, we discuss natural activators and potential therapeutic strategies targeting Prdx6 to extend eye lens health and delay or prevent cataract development. Overall, we conclude that enhancing Prdx6 activity offers a promising strategy to prevent or reverse age-related cataracts.

## 1. Introduction

**Fig. 1.**
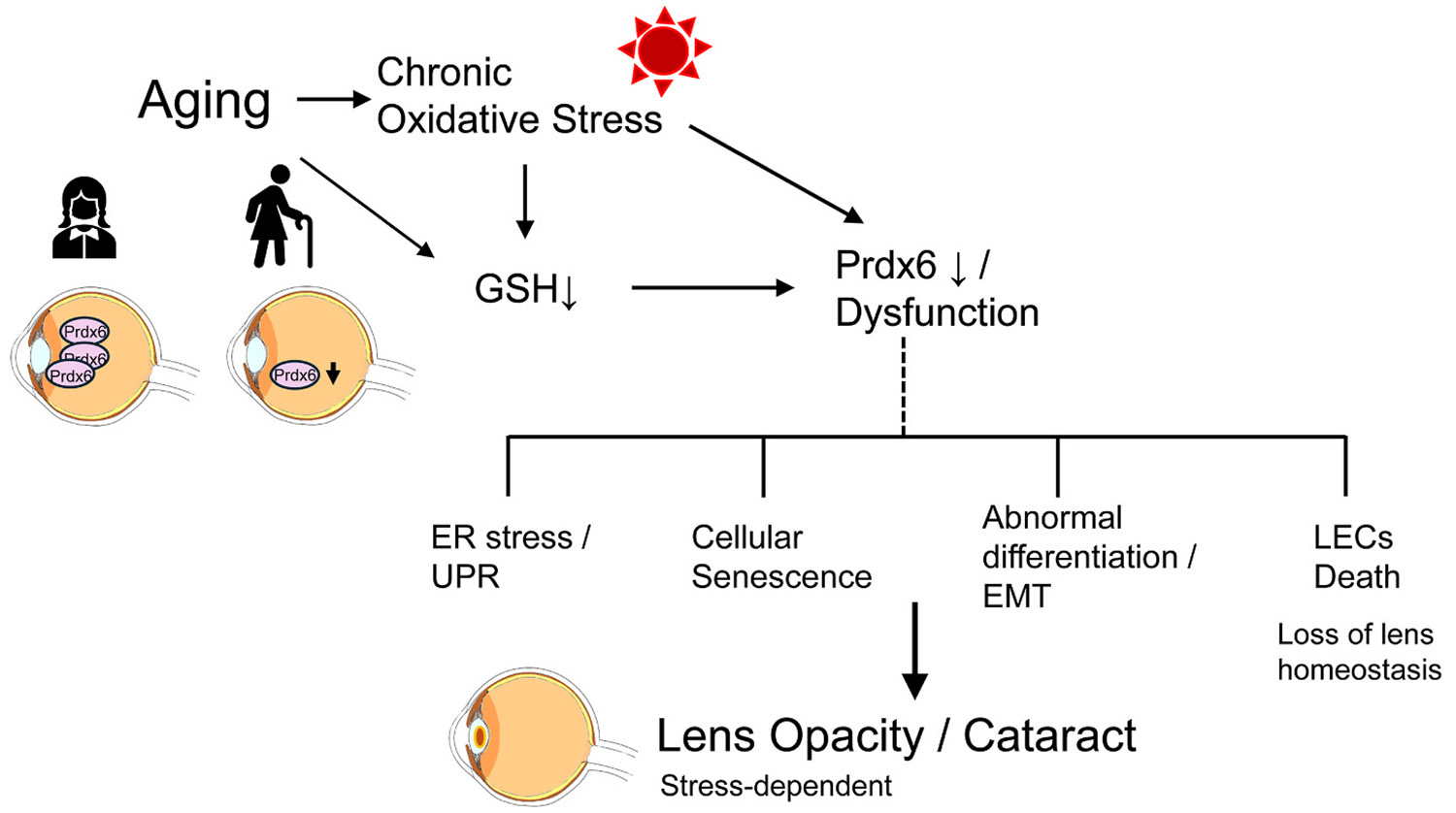
Integrated model of aging-associated oxidative stress and Prdx6-dependent lens pathology. Aging is accompanied by chronic oxidative stress and a progressive decline in glutathione, leading to reduced or dysfunctional Prdx6 and impaired redox homeostasis in lens epithelial cells. Loss of Prdx6 function sensitizes the lens to oxidative damage and triggers stress-dependent cellular responses, including endoplasmic reticulum stress/unfolded protein response, cellular senescence, abnormal differentiation/epithelial–mesenchymal transition, and lens epithelial cell death with loss of lens homeostasis. These interconnected processes collectively contribute to lens opacity and age-related cataract formation in a stress-dependent manner. Abbreviations: Prdx6, peroxiredoxin 6; GSH, glutathione; LECs, lens epithelial cells; ER stress/UPR, endoplasmic reticulum stress/unfolded protein response; EMT, epithelial–mesenchymal transition.

Reactive oxygen species (ROS) are highly reactive natural byproducts of cellular metabolism and play essential roles in cell signaling (D'Autreaux and Toledano, 2007; Finkel, 2011). Excessive ROS production, particularly in response to defective metabolic machinery such as mitochondrial activity or environmental stressors, can disrupt the cell's antioxidant defense mechanisms, leading to cellular damage. ROS are excessively generated when an imbalance occurs between their production and antioxidant elimination; this imbalance may arise from endogenous and exogenous stressors or weak antioxidant responses (Balaban et al., 2005; Dai et al., 2014; El Assar et al., 2013; Finkel and Holbrook, 2000; Franco et al., 2009; Hybertson et al., 2011). Consequently, they may damage cellular components such as proteins, lipids, and nucleic acids, leading to disrupted cellular homeostasis and an array of cell death signaling pathways, including aberrant post-translational modifications (PTMs) (Chhunchha et al., 2022a, 2022b; Finkel, 2011; Hayes and Dinkova-Kostova, 2014; Kubo et al., 2008, 2017; McCormick and Promislow, 2018; Shibata et al., 2016). Moreover, excessive oxidative stress-induced cell death and damage driven by elevated ROS levels are major contributors to numerous pathological conditions, including neurodegenerative diseases, cardiovascular disorders, cataracts, and other aging-associated conditions (Franco and Cidlowski, 2009; Spector, 2000; Valko et al., 2006, 2007). Recent studies have revealed molecular crosstalk among apoptosis, pyroptosis, necroptosis, and ferroptosis. These cell death modalities have been identified in aging lens epithelial cells (LECs) subjected to oxidative stress (Ai et al., 2024; Bertheloot et al., 2021; Eskander et al., 2025; Hong et al., 2017; Pacheco, 2024; Sorkou et al., 2023; Wei et al., 2021) and have been implicated in the onset of cataracts (Guo et al., 2025; Huang et al., 2025; Li et al., 2024; Osnes-Ringen et al., 2016; Sheng et al., 2024; Wei et al., 2021). However, oxidative stress and aging share common molecular pathways and are major initiating factors in the pathogenesis of various age-related diseases (ARDs), including blinding disorders (Chhunchha et al., 2022a, 2022b; Dai et al., 2014; Shaw et al., 2014). Emerging evidence suggests that either delaying or treating a single ARD may confer resistance or resilience against other aging-related pathologies (Franceschi et al., 2018a, 2018b; Mercken et al., 2017). Importantly, this concept is supported by direct evidence from human anterior lens capsule tissues. Analyses of LECs obtained from anterior capsule specimens during human cataract surgery demonstrated an age-dependent decline in antioxidant defense, including peroxiredoxin 6 (Prdx6) expression, which inversely correlated with the severity of nuclear and cortical age-related cataracts (Hasanova et al., 2009; Pak et al., 2006). These findings indicate that aging-associated oxidative stress in human lens epithelial cells is directly relevant to age-related cataract formation. An integrated overview of aging-associated oxidative stress, Prdx6 dysfunction, and stress-dependent cataractogenesis is shown in Fig. 1.

Molecular investigations into oxidative stress, redox signaling, and different modalities of cell death versus survival have highlighted the roles of Prdx6, also known as antioxidant protein 2 (AOP2), nuclear factor erythroid 2-related factor 2 (Nrf2), and selenoproteins such as glutathione peroxidase 4 (GPx4) and GPx1. These molecules participate in redox regulation and play key roles in determining cell fate during disease progression, including cataracts (Chhunchha et al., 2014, 2017, 2018, 2019, 2020, 2023a, 2023b; Fujita et al., 2024; Ito et al., 2024; Jin et al., 2018; Lopez-Grueso et al., 2020; Lu et al., 2019; Minutoli et al., 2016; Nishizawa et al., 2023; Patel et al., 2014; Singh et al., 2023; Sun et al., 2020; Zucker et al., 2014). Furthermore, proteins can have differential susceptibility to ROS-driven modifications, such as Small Ubiquitin-like Modifier (SUMO) conjugation (SUMOylation), phosphorylation, and acetylation (Feligioni and Nistico, 2013; Ray et al., 2015). Thus, ROS-induced aberrant protein modifications are also involved in the pathology of several human diseases (Chhunchha et al., 2014; Flotho and Melchior, 2013). However, Prdxs, including Prdx6, act as sensor molecules for cellular H_2_O_2_ and are directly involved in modulating signaling pathways (Jarvis et al., 2012; Sobotta et al., 2015). Controlled levels of hydrogen peroxide (H_2_O_2_), ranging from 1 nM to 100 nM, are required for normal physiological functioning of cells, and the effects of ROS can vary based on cell types and cellular microenvironment (Sies, 2017, 2023; Sies et al., 2017, 2024; Sies and Jones, 2020).

**Fig. 2.**
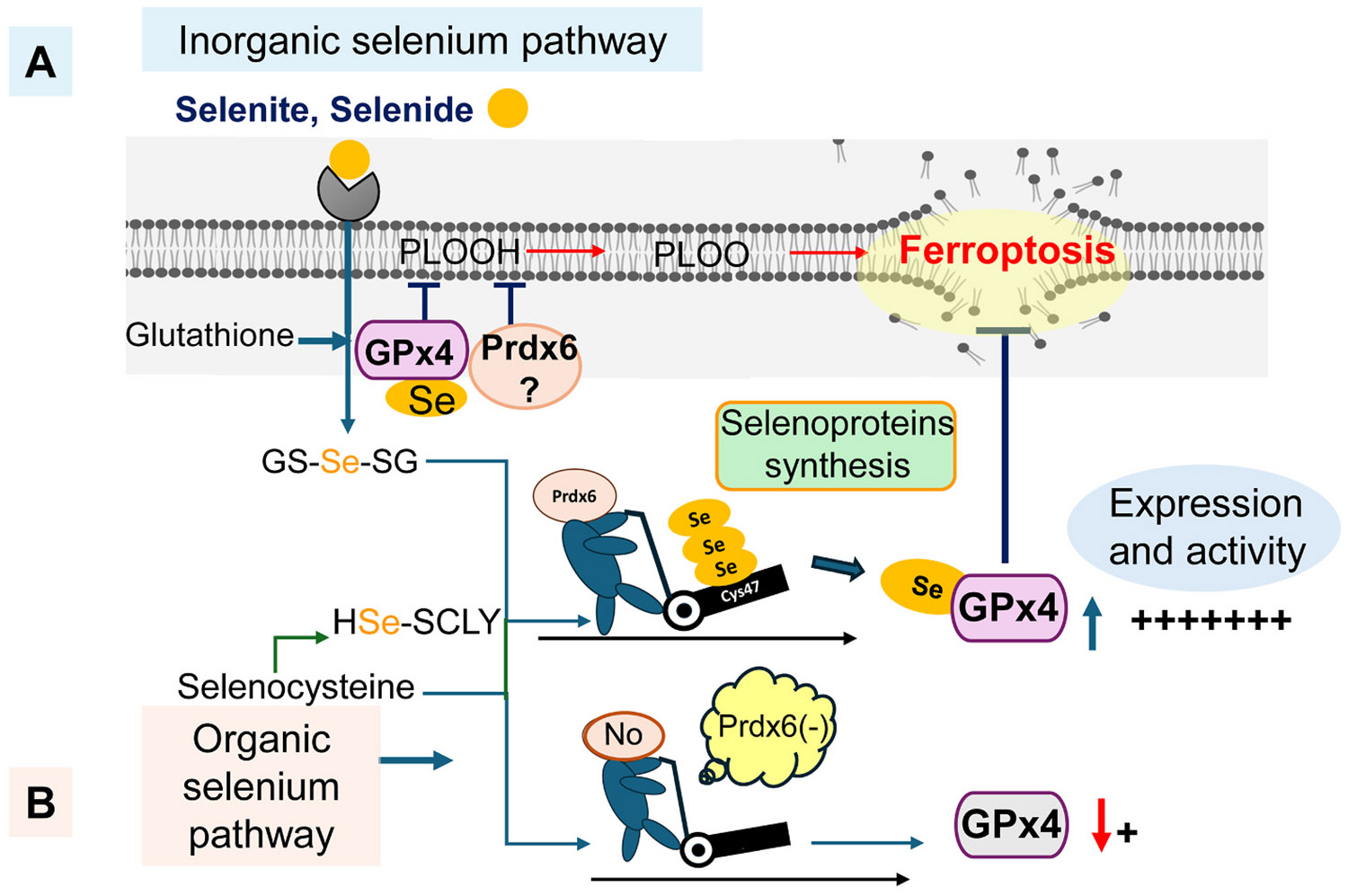
Functional interplay between Prdx6 and selenoproteins in intracellular selenium handling and ferroptosis prevention. Beyond its established trifunctional enzymatic activities, Prdx6 functionally cooperates with the selenoprotein biosynthesis machinery to support cellular redox homeostasis. (A) In the inorganic selenium pathway, intracellular selenium may interact with Prdx6 at the active-site cysteine in a glutathione-dependent manner, forming a transient Prdx6–selenium complex that contributes to selenium availability for selenoprotein synthesis, including GPx4. (B) In the organic selenium pathway, selenium released via the selenocysteine lyase pathway is utilized by selenophosphate synthetase 2 for selenoprotein biosynthesis. Through this coordinated process, Prdx6 indirectly supports GPx4 function and limits ferroptotic cell death. Prdx6 deficiency is associated with reduced expression and activity of selenoproteins, resulting in increased susceptibility to lipid peroxidation–driven cell death. Abbreviations: Prdx6, peroxiredoxin 6; GPx4, glutathione peroxidase 4; GSH, glutathione; PLOOH, phospholipid hydroperoxide; PLOO·, lipid peroxyl radical; SCLY, selenocysteine lyase.

Prdxs are small antioxidant proteins (20–30 kDa) that account for approximately 1 % of the total soluble cellular proteins (Manevich et al., 2002, 2007; Seo et al., 2000; Wood et al., 2003). Prdx6 is a unique member of the peroxiredoxin family (Prdx1–6) that is distinguished by both its structural and functional properties. Unlike the typical 2-Cysteine (Cys) Prdx1–5 isoforms, which rely on the thioredoxin (Trx) system for catalytic recycling, Prdx6 contains a single catalytic cysteine (Cys47) and is regenerated independently of Trx, utilizing the glutathione (GSH) system instead (Fisher, 2011; Manevich and Fisher, 2005). This Trx-independent mechanism is particularly relevant in aging and oxidative stress conditions, where Trx system activity is compromised. Through this complementary redox strategy, Prdx6 cooperates with Trx-dependent antioxidant systems to maintain redox homeostasis in the lens. Prdx6 provides an alternative and resilient antioxidant defense, particularly under conditions of elevated oxidative stress. In addition to its glutathione peroxidase activity, Prdx6 is a multifunctional protein exhibiting acidic calcium-independent phospholipase A_2_ (aiPLA_2_) and lysophosphatidylcholine acyltransferase (LPCAT) activities, further contributing to membrane repair and cellular homeostasis in lens epithelial cells (Arevalo and Vazquez-Medina, 2018). Recently, Prdx6 has been demonstrated to facilitate selenium mobilization for selenoproteins, including GPx4, and exert lipid peroxidation–related activity via GPx4 (Chen et al., 2024; Fujita et al., 2024; Ito et al., 2024). Prdx6 depletion leads to a significant decrease in selenoprotein expression and triggers ferroptotic cell death due to a reduction in GPx4 levels, whereas overexpression of Prdx6 reverses this process. Thus, Prdx6 is pivotal in regulating ferroptosis by directing selenium mobilization and facilitating selenoproteins, including GPx4, the most prominent regulator of ferroptosis, which prevents phospholipid peroxidation by reducing phospholipid hydroperoxides (PLOOHs) to their related alcohols (Fig. 2) (Chen et al., 2024; Fujita et al., 2024; Ito et al., 2024).

This review aimed to provide a comprehensive overview of Prdx6's physiological roles in redox biology, its regulation of selenium metabolism, and its interaction with selenoproteins. We also explored its involvement in regulating and limiting ROS and PLOOHs and modulating inflammatory pathways in the eye lens. By exploring the significance of Prdx6 in eye lens biology and the molecular mechanisms underlying the protective effects of Prdx6, this review highlights potential therapeutic strategies for the management of age-related cataracts.

## 2. Prdx6 protection mechanism and its interplay with selenoproteins

1-Cys Prdx6 is a unique member of the Prdx family, distinguished by its pleiotropic protective activity in maintaining cellular homeostasis. Notably, it functions as an antioxidant independently of other peroxiredoxins and classical thiol-dependent antioxidant systems. (Chhunchha et al., 2017, 2022b, 2023b; Eismann et al., 2009; Fatma et al., 2011; Fisher et al., 2016; Manevich et al., 2002; Sorokina et al., 2011; Wang et al., 2003). Prdx6 safeguards cells, tissues, and organs, and its presence is essential for cellular health and protection against ROS- and lipid peroxidation–mediated damage to membranes, DNA, and proteins (Abais et al., 2015; Bolduc et al., 2021; Kubo et al., 2008). Prdx6 deficiency leads to ROS accumulation, resulting in elevated oxidative stress, cellular abnormalities, and cataract formation in *Prdx6*^*−/−*^ LECs and lenses, which is reversed by exogenous Prdx6 delivery (Fatma et al., 2005; Kubo et al., 2008, 2009).

**Fig. 3.**
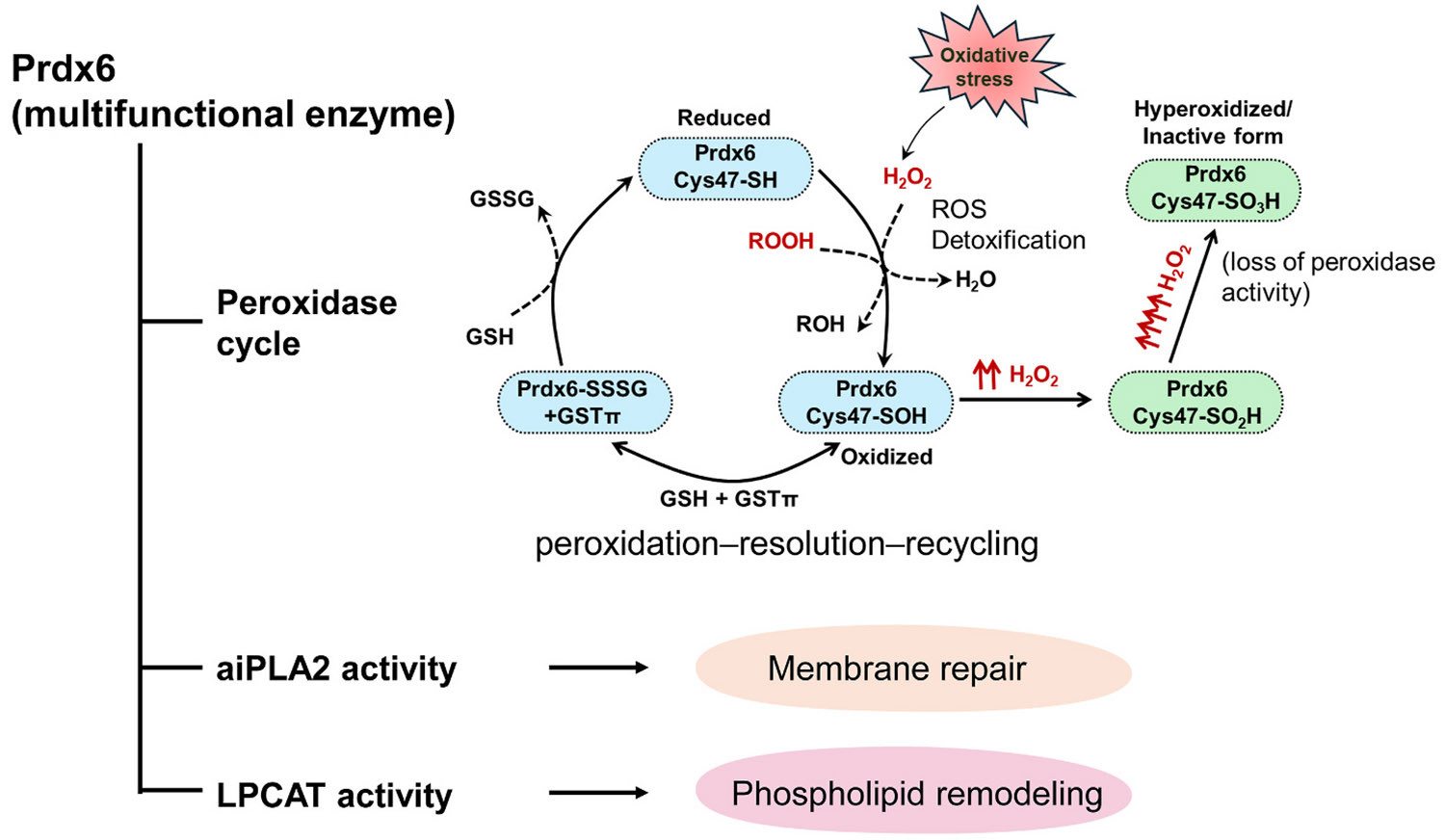
Integration of Prdx6 enzymatic activities with physiological functions. Prdx6 is a multifunctional enzyme exhibiting glutathione peroxidase, acidic calcium-independent phospholipase A2, and lysophosphatidylcholine acyltransferase activities. The peroxidase activity of Prdx6 proceeds through a three-step catalytic cycle—peroxidation, resolution, and recycling—mediated by redox cycling of the active-site cysteine and the glutathione/GSTπ system. Under excessive oxidative stress, the sulfenic acid form of the active-site cysteine can be hyperoxidized to sulfinic or sulfonic forms, resulting in loss of peroxidase activity. In parallel, the phospholipase and acyltransferase activities contribute to membrane repair and phospholipid remodeling, respectively. Through these coordinated enzymatic functions, Prdx6 maintains redox balance, membrane integrity, and cellular homeostasis under oxidative stress conditions. Abbreviations: Prdx6, peroxiredoxin 6; aiPLA2, acidic calcium-independent phospholipase A2; LPCAT, lysophosphatidylcholine acyltransferase; GSH, glutathione.

Mechanistically, the peroxidase activity of Prdx6 consists of three main steps: peroxidation, resolution (reduction of cysteine sulfenic acid), and recycling (regeneration of the cysteine active site), as illustrated in Fig. 3. These steps rely on GSH and glutathione S-transferase (GST) for their completion (Fisher, 2011). Importantly, Prdx6 reduces fatty acid hydroperoxides to their corresponding free fatty acids and phospholipid components, thereby protecting cells from oxidative damage caused by lipid peroxidation. In addition to its peroxidase activity, Prdx6 exhibits phospholipase A_2_ (PLA_2_) activity, which is responsible for hydrolyzing phospholipids and plays a key role in membrane repair (Manevich et al., 2007, 2009). This enzymatic activity enables Prdx6 to repair oxidized cell membranes by removing damaged lipids and replacing them with healthy lipids, such as monounsaturated and polyunsaturated fats. LPCAT, which is closely linked to PLA_2_, further contributes to membrane repair by catalyzing the reaction of lysophospholipids with palmitoyl-CoA (Kakchingtabam et al., 2025; Rahaman et al., 2024). Prdx6 exhibits pH-dependent aiPLA_2_ activity that arises from the formation of an oligomeric state at low pH. This oligomer is highly stable and more thermotolerant than the protein at normal pH (7.4) (Chowhan et al., 2021), thereby conferring aiPLA_2_ activity and cell membrane protection (Kakchingtabam et al., 2025).

Moreover, as a non-selenoprotein, the unique properties of Prdx6 have recently been attributed to synergistic interactions with selenoproteins. Prdx6 is involved in selenium metabolism and contributes to the expression and activity of selenoproteins, such as GPx4 and GPx1, by facilitating selenium availability, which is a prerequisite for their expression and function (Chen et al., 2024; Fujita et al., 2024; Ito et al., 2024). Previously, the protective activities of Prdx6 were believed to be fully independent. However, recent studies have revealed that the activities of Prdx6, which are similar to those of GPx4 in limiting a wide range of peroxides, including H_2_O_2_, PLOOHs, and reactive nitrogen species like peroxynitrite (Fisher, 2011; Fisher et al., 1999; Li et al., 2024; Manevich and Fisher, 2005; Manevich et al., 2002), are dependent on GPx4 and vice versa (Chen et al., 2024; Fujita et al., 2024; Ito et al., 2024). These studies demonstrate that Prdx6, although not a selenoprotein, functionally cooperates with selenoproteins, such as GPx4, to sense and regulate cellular redox homeostasis. Through this coordinated action, Prdx6 and GPx4 contribute to the prevention of aberrant cell death signaling and protect cells against environmental stress and metabolic imbalance (Chen et al., 2024; Fujita et al., 2024; Ito et al., 2024) (Fig. 2). Although intracellular selenium was considered to be supplied solely by carrier biomolecules (Fujita et al., 2024), Prdx6 is now presumed to be involved as a novel factor involved in GPx4 synthesis. Cys47 in Prdx6 is critical for selenium facilitation required for the expression of selenoprotein GPx4 (Ito et al., 2024). Ito et al. (2024) subsequently demonstrated that peroxidative Cys47 is a prerequisite for selenium handling as well as physiological expression and activity of selenoproteins such as GPx4. Furthermore, a recent study using *Prdx6*-deficient LECs and lenses corroborated that Prdx6 is required for GPx4 expression and its protective activity (Singh et al., 2025). Notably, even in Prdx6-deficient lung endothelial cells, ferroptosis susceptibility persists despite a compensatory increase in GPx4 levels (Torres-Velarde et al., 2024), suggesting the importance of Prdx6 in blocking the ferroptosis pathway (Fig. 2). Although the above studies suggest a critical role of Prdx6 in regulating selenoprotein expression and activity, several aspects warrant further investigation. For instance, Prdx6 does not possess deselenase activity; thus, elucidating how selenium is released from selenocysteine is crucial.

## 3. Aging, oxidative stress, and Prdx6 as an integrative regulator of lens pathology

### 3.1. Expression of Prdx6 and eye lens integrity and cataractogenesis

Aging-associated oxidative stress represents a fundamental driver of LECs dysfunction and cataractogenesis. Accumulating evidence indicates that Prdx6 occupies a central position in this process by integrating redox regulation, cellular stress responses, and lens homeostasis.

Age-dependent decline or functional impairment of Prdx6 compromises antioxidant defense, amplifies oxidative stress, and sensitizes lens epithelial cells to downstream pathological events, including endoplasmic reticulum (ER) stress, cellular senescence, abnormal differentiation, and cell death. Thus, Prdx6 functions as a critical molecular node linking aging-related oxidative damage to the initiation and progression of lens pathology. Prdx6 is present in nearly all major organs, including ROS-producing cellular components such as the mitochondria, ER, and the plasma membrane, to maintain redox signaling and cell health (Eismann et al., 2009; Fisher, 2011; Kubo et al., 2017; Manevich et al., 2014). However, previous studies have demonstrated that Prdx6 is localized in the cytoplasm of LECs and lens fiber cells, with dynamic developmental and age-dependent expression patterns (Chhunchha et al., 2020, 2022b, 2023b; Detcheverry et al., 2023; Ferrer et al., 1990; Kubo et al., 2006). In situ hybridization and immunohistochemical analyses have revealed that Prdx6 is localized in the cytoplasm of LECs and lens fiber cells. Intense Prdx6 staining was observed throughout the lens on gestational days 14 and 18. In contrast, lenses from postnatal day 1 mice showed diminished nuclear fiber staining, whereas those from 4-week-old mice showed a lack of nuclear fiber staining but intense staining in the germinative zone (an active LEC proliferation site with increased ROS production). Prdx6 expression gradually increased in the lenses of 4-week- to 6-month-old mice and then declined (Kubo et al., 2006). Notably, GSH is an electron donor for the revival of oxidized Prdx6 under oxidative stress (Manevich and Fisher, 2005). This parallel age-dependent decline in both Prdx6 and GSH likely synergistically impairs Prdx6-mediated antioxidant capacity in the aging lens, thereby exacerbating oxidative stress and increasing susceptibility to cataract formation (Ferrer et al., 1990; Kubo et al., 2006; Lou, 2003; Manevich and Fisher, 2005). Both Prdx6 and GSH decline with aging, suggesting that this event can be related to the development of cataractogenesis due to the accumulation of ROS-driven oxidative load in aging (Chhunchha et al., 2020, 2022b, 2023b; Detcheverry et al., 2023; Ferrer et al., 1990; Kubo et al., 2006) (Fig. 1). In the previous section, we discussed that Prdx6 is an essential element in maintaining cellular and organ health in various tissues, including the eye lens (Fatma et al., 2005; Fisher, 2011; Fisher et al., 2016; Wang et al., 2003), and its protective functions are dependent on its level of expression. A decline in Prdx6 expression is strongly associated with lens cell death and the severity of lens opacity in response to oxidative stress (Chhunchha et al., 2022a, 2022b; Fatma et al., 2005; Kubo et al., 2006, 2008). This suggests that reduced Prdx6 levels accompanying aging and cataract progression may underlie the increased oxidative damage in cataractous lenses. A strong correlation between Prdx6 expression, patient age, and cataract severity has been reported (Hasanova et al., 2009). Importantly, human lenses with advanced nuclear and cortical cataracts also show significantly lower Prdx6 levels than those in clear lenses (Kubo et al., 2006), and loss of Prdx6 is strongly correlated with the severity of cataracts. In addition, Prdx6 deficiency and oxidative stress have been linked to the development of cellular senescence and age-related diseases, including cataractogenesis (Qin et al., 2025; Wang et al., 2025). In vitro, ex vivo, and in vivo studies have demonstrated that LECs undergo senescence, which contributes to cataract initiation and progression and can be attenuated by the induction of antioxidants (Chen et al., 2021, 2022; Cooksley et al., 2024; Yan et al., 2019; Ye et al., 2022). Mechanistically, accumulating evidence indicates that Prdx6 plays a critical role in maintaining ER homeostasis under oxidative and hypoxic stress conditions. Loss of Prdx6 induces ER stress and activates the unfolded protein response (UPR), leading to increased oxidative burden, impaired cellular homeostasis, and cellular damage in LECs as well as in other cell types. In contrast, restoration or supplementation of Prdx6 attenuates oxidative and ER stress, including hypoxia-driven ER dysfunction (Asuni et al., 2015; Chhunchha et al., 2013; Fatma et al., 2011). Importantly, Prdx6 is a well-recognized target of oxidative stress–driven PTMs, such as aberrant SUMOylation and overoxidation of its catalytic cysteine residue, which compromise Prdx6 stability and enzymatic activity (Chhunchha et al., 2014, 2017; Shibata et al., 2016). PTM-mediated loss of Prdx6 function further amplifies redox imbalance and ER stress/UPR signaling, creating a cellular environment that favors abnormal protein folding and proteostasis failure in LECs. Although Prdx6 is not a classical protein-folding chaperone, its role in regulating intracellular redox homeostasis and ER stress places it at a critical interface between oxidative stress, aberrant protein modification, and protein misfolding–associated lens pathology. Furthermore, the lens resides in a physiologically hypoxic environment, and hypoxia-associated metabolic stress exacerbates ER stress and UPR activation in LECs, promoting oxidative damage and cataract formation (Beebe et al., 2014; Elanchezhian et al., 2012a, 2012b; Palsamy et al., 2012).

Notably, visible lens opacities were not observed in Prdx6-knockout (*Prdx6* ^*−/−*^) mice (Fatma et al., 2005). This phenotype may be explained, at least in part, by the fact that Prdx6-deficient mice were maintained under relatively unstressed physiological conditions and were not subjected to chronic environmental or metabolic stress. Under these basal conditions, loss of Prdx6 alone appears insufficient to trigger spontaneous lens opacity. In contrast, exposure to oxidative stress markedly increases lens vulnerability in Prdx6-deficient models, suggesting that cumulative oxidative insults act as critical triggers for cataractogenesis in aging lenses, in which Prdx6 levels are reduced (Kubo et al., 2006; Fatma et al., 2005).

Consistent with this stress-dependent vulnerability, a closer examination of *Prdx6* ^*−/−*^ lenses revealed abnormalities in fiber cell differentiation and LECs migration, including defective bow region formation (Fatma et al., 2005; Kubo et al., 2010). Notably, *Prdx6* ^*−/−*^ LECs exhibit enhanced susceptibility to H_2_O_2_-induced cell death, and Prdx6-deficient lenses develop opacities at significantly lower oxidative stress levels and shorter exposure durations than controls (Fatma et al., 2005). Moreover, large-scale proteomic and gene expression analyses have demonstrated substantial dysregulation of multiple pathways, including the marked upregulation of cytoskeletal proteins such as tropomyosin and vimentin, further supporting the causal role of Prdx6 loss in sensitizing the lens to oxidative stress–driven pathology (Kubo et al., 2010). Additionally, transforming growth factor-beta 2 (TGF-β2), a key factor in cell differentiation, was upregulated in *Prdx6* ^*−/−*^ LECs (Fatma et al., 2005; Kubo et al., 2010). TGF-β2 treatment also induced tropomyosin expression in control mouse and rat LECs (Kubo et al., 2010, 2013), promoted epithelial-mesenchymal transition (EMT), and was associated with cataract formation and posterior capsule opacification in rats (Kubo et al., 2013). These findings suggest that the morphological and differentiation abnormalities in *Prdx6*^*−/−*^ mouse lenses are a result of changes in the expression of genes such as TGF-β2 and tropomyosin, which are associated with EMT in LECs (Kubo et al., 2010, 2013).

Despite the strong association between reduced Prdx6 expression and age-related cataract (ARC) severity, it is important to distinguish correlation from causality, particularly in human samples. Most evidence linking Prdx6 decline to cataractogenesis in patients is derived from cross-sectional analyses of surgical specimens, which cannot definitively establish whether reduced Prdx6 expression represents a primary pathogenic driver or a secondary consequence of chronic oxidative stress and cellular injury (Hasanova et al., 2009).

Importantly, *Prdx6* ^*−/−*^ mice do not develop spontaneous cataracts under stress-free physiological conditions, indicating that the loss of Prdx6 alone is insufficient to induce lens opacity (Fatma et al., 2005). Rather, Prdx6 deficiency confers increased vulnerability to oxidative and metabolic stress, under which lens epithelial cells exhibit enhanced cell death and accelerated cataract-related changes, including increased sensitivity to H_2_O_2_-induced injury (Fatma et al., 2005; Kubo et al., 2008). This conditional phenotype underscores the critical role of environmental and lifetime stress exposure in triggering cataractogenesis and provides a framework that better aligns experimental models with human ARC, which develops through cumulative oxidative insults over decades. Furthermore, although correlations between Prdx6 expression, aging, and cataract severity have been reported, systematic studies directly linking Prdx6 levels with quantitative oxidative stress markers and molecular aging indicators in human lenses are limited (Lou, 2003; Spector, 2000). Addressing these gaps through longitudinal and integrative analyses is essential to define the precise causal contribution of Prdx6 to human cataract pathogenesis.

### 3.2. Role of PTMs of Prdx6 in cellular physiology

Prdx6 is a multitasking protein that performs several activities that promote cellular health. PTMs play an important role in maintaining quality control of genetically encoded protein functions. As a multifunctional protein, Prdx6 undergoes several PTMs that are involved in its intended function at physiological levels and in response to oxidative stress. In addition to these modifications, Prdx6 is also susceptible to other oxidative stress–associated PTMs commonly observed in aging tissues, including ubiquitination, non-enzymatic glycation, and aldehyde adduction, although their specific functional consequences for Prdx6 remain incompletely defined. In response to oxidative stress, Prdx6 can undergo several PTMs, which can be identified using proteomic tools, including nano–ultra-performance liquid chromatography-–electrospray ionization–quadrupole time-of-flight tandem mass spectrometry (nanoUPLC-ESI-q-TOF MS/MS) in conjunction with the MODi and MODmap algorithms (Jeong et al., 2012). This analysis indicated that Prdx6 undergoes several PTMs, including phosphorylation, deamidation, acetylation, SUMOylation, sulfenic/sulfonic acids at cysteine 47, S-Palmitoylation, and that these PTMs are involved in Prdx6 regulation and function (Jeong et al., 2012). However, the functional roles of all PTMs remain unknown. Herein, we briefly discuss some important PTMs, the functions of which are established in the regulation and regulatory role of Prdx6, and which should be helpful in understanding how Prdx6 may play key roles under oxidative stress. Moreover, oxidative stress-induced aberrant protein modification has been implicated in the etiology and progression of several human diseases (Anderson et al., 2017; Feligioni and Nistico, 2013; Ray et al., 2015; Vijayakumaran and Pountney, 2018). Maintaining Prdx6 activity is crucial for lens health, as it mitigates oxidative damage, prevents the aggregation of oxidized proteins, and supports cell survival in the lens. PTMs are chemical alterations in proteins that occur after their synthesis and can affect protein function, stability, and localization. Aging is associated with increased PTMs, many of which negatively impact activity and compromise the lens's defense against oxidative stress. Below, we present a few key PTMs that affect Prdx6 expression during aging. These PTMs, which accumulate with aging and chronic oxidative stress, may cooperatively modulate Prdx6 stability, turnover, and activity, thereby influencing its protective capacity in the lens. Further systematic characterization of these age-associated PTMs is essential to fully understand how Prdx6 function is altered in aging and cataractous tissues.

#### 3.2.1. SUMOylation

SUMOylation is the attachment of SUMO proteins to target proteins, regulating their stability and activity (Anderson et al., 2017). Under oxidative stress, Prdx6 is aberrantly SUMOylated by SUMO1, resulting in the loss of its antioxidant function and promotion of cellular damage (Chhunchha et al., 2014, 2017). Lysine residues K122 and K142 were identified as the major SUMO1-binding sites in Prdx6. A SUMOylation-deficient mutant (Prdx6K122/142R) shows increased stability, enhanced enzymatic activities, and improved cytoprotection (Chhunchha et al., 2017; Kubo et al., 2008). SUMOylation also disrupts Prdx6's PLA_2_ activity, further reducing its capacity to repair oxidized membranes (Chhunchha et al., 2017). The increased protective activity of SUMOylation-deficient Prdx6 has been linked to elevated GSH peroxidase and PLA_2_ activities compared with those of wild-type Prdx6 (Prdx6WT) (Chhunchha et al., 2017). These findings suggest that targeting SUMOylation sites may offer a novel therapeutic strategy for the treatment of oxidative stress-related diseases. Importantly, ROS-induced aberrant SUMOylation drastically reduces Prdx6 stability and function, contributing to cell death—an insight potentially relevant to many degenerative diseases. In addition to direct SUMOylation of Prdx6, SUMOylation of transcriptional regulators such as Sp1 also contributes to age- and stress-dependent suppression of Prdx6 expression, as discussed in Section 4.3.

#### 3.2.2. Phosphorylation

Phosphorylation is a common PTM that regulates protein activities. For Prdx6, phosphorylation at threonine 177 (T177) is critical for its PLA_2_ function and antioxidant defense. Mutations at this site significantly reduce Prdx6 enzymatic activity, underscoring T177's importance in regulating protective functions (Chhunchha et al., 2017). Proper phosphorylation enables Prdx6 to perform both its GPx and PLA_2_ activities—key for neutralizing ROS and repairing oxidized membranes. Impaired phosphorylation compromises Prdx6's repair capacity, weakening the lens's defense against oxidative stress and promoting cataract formation.

#### 3.2.3. Oxidation

Prdx6 is particularly susceptible to oxidation at its catalytic cysteine residue (Cys47), which is essential for peroxidase activity (Fatma et al., 2001; Fisher, 2011). During aging, elevated oxidative stress leads to overoxidation of Cys47 into cysteine sulfinic or sulfonic acid (Shibata et al., 2016; Wagner et al., 2002; Woo et al., 2003; Yang et al., 2002). This irreversible modification inactivates Prdx6, preventing it from neutralizing hydrogen peroxide and other ROS. Accumulation of oxidized, inactive Prdx6 diminishes antioxidant protection, accelerating cataract formation (Shibata et al., 2016).

## 4. Regulatory pathway of Prdx6 expression in the lens

**Fig. 4.**
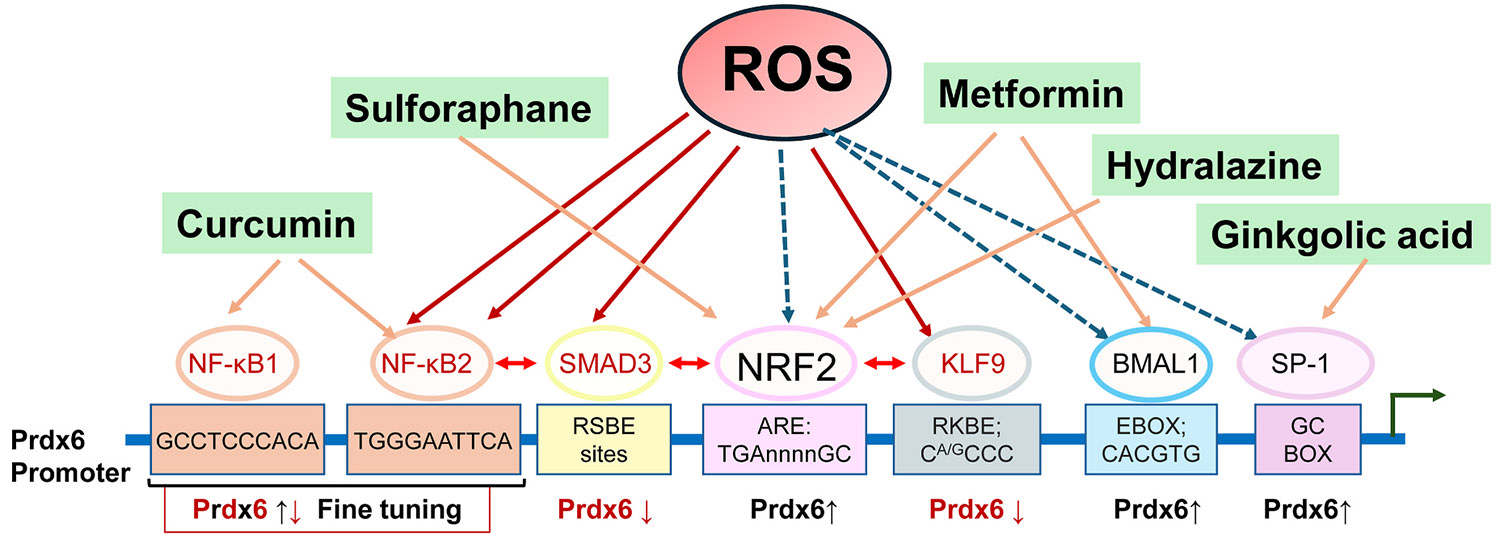
Transcriptional regulation of Prdx6 expression by multiple transcription factors. Reactive oxygen species generated during aging or oxidative stress modulate Prdx6 transcription through the coordinated action of multiple transcription factors, including NF-κB, SMAD3, NRF2, KLF9, Bmal1, and Sp1, which bind to distinct regulatory elements within the Prdx6 promoter. Abbreviations: Prdx6, peroxiredoxin 6; ROS, reactive oxygen species; NF-κB, nuclear factor-κB; NRF2, nuclear factor erythroid 2–related factor 2.

As mentioned above, the expression of Prdx6 is almost ubiquitous across all mammalian organs and tissues, but it is highly expressed in oxidative stress-facing cells and organelles (Chhunchha et al., 2017, 2020; Fisher, 2011; Kubo et al., 2017; Manevich et al., 2002). However, the expression of Prdx6 varies across different tissues and cell types, showing elevated levels in lens/LECs and lung epithelial cells, among others (Chen et al., 2000; Fisher, 2011; Kubo et al., 2006; Manevich et al., 2002). This discrepancy may arise from the diverse stimulation of transcription factors involved in the regulation of Prdx6 transcription within the cellular microenvironment and cell types (Ambruso et al., 2012; Patel and Chatterjee, 2019). Prdx6 is modulated by various factors, including ROS-responsive transcription factors such as Nrf2, hypoxia-inducible factor (HIF), nuclear factor-κB (NF-κB), activator protein 1 (Ap-1), c-myelocytomatosis oncogene (c-Myc), and Krüppel--like factor 9 (Klf9), as well as other transcriptional regulators and immune modulators. (Fatma et al., 2009a; Kubo et al., 2017; Sharapov et al., 2019) (Fig. 4). Moreover, reportedly, H_2_O_2_-driven excessive oxidative stress can result in a significant decrease in Prdx6 levels (Chen et al., 2024; Chhunchha et al., 2022a; Fatma et al., 2009a). Under normal conditions, Nrf2, a key regulator of Prdx6, binds to kelch-like ECH-associated protein 1 (Keap1) in the cytoplasm—targeting it for degradation (Itoh et al., 1999; Suzuki and Yamamoto, 2015). During oxidative stress, Nrf2 dissociates from Keap1, translocates to the nucleus, and binds to antioxidant response elements (AREs) in the promoter of the *Prdx6* gene (ARE sequence position: 357 to −349). The Nrf2/ARE pathway is critical for cellular protection against oxidative damage, particularly in aging and diseases such as cancer and cataracts, with oxidative stress being implicated in the etiology of aging-related disorders (Chhunchha et al., 2014; Chhunchha et al., 2021; DeNicola et al., 2011; Jaramillo and Zhang, 2013; Kubo et al., 2017; O'Connell and Hayes, 2015; Palsamy et al., 2012; Palsamy et al., 2014). In aging human LECs, the Nrf2/ARE pathway becomes inactive, leading to the loss of Prdx6 expression and oxidative stress-induced cell death (Chhunchha et al., 2019, 2023b; Kubo et al., 2017) (Figs. 1 and 4). Consistent with this regulatory framework, genetic disruption of Nrf2 has been shown to markedly impairs antioxidant defense in ocular tissues, including the lens, resulting in increased susceptibility to oxidative stress–induced damage (Chang et al., 2022; Chhunchha et al., 2020; DeNicola et al., 2011; Kubo et al., 2017). Given that Prdx6 is a downstream target of Nrf2, these findings provide in vivo genetic support for the functional relevance of the Nrf2–Prdx6 axis in maintaining lens homeostasis. Moreover, Prdx6 is a target for the redox-active nuclear protein NF-*κ*B, and its expression is modulated by NF-*κ*B under conditions of oxidative stress and in redox-active or aging LECs (Chhunchha et al., 2013; Fatma et al., 2009b; Singh et al., 2016). NF-*κ*B plays multiple roles in cell physiology by regulating survival and cell death pathways (Piette et al., 1997; Schreck and Baeuerle, 1991). Using redox-active, Prdx6-deficient LECs, we showed that activated NF-*κ*B significantly dysregulates Prdx6 transcription by binding to its site in the Prdx6 promoter (NF-*κ*B site: 5′-GTGGGAATTCA-3′), suggesting that NF-*κ*B acts as a suppressor of Prdx6 expression (Fatma et al., 2009a). In aging cells/LECs, NF-*κ*B is activated and drives cell death signaling (Gorgoulis et al., 2019; Salminen et al., 2008; Tilstra et al., 2011; Zhang et al., 2017). We surmise that in aging lenses/LECs, activated NF-*κ*B suppresses Prdx6 expression, leading to ROS amplification-mediated lens pathobiology (Fig. 4).

### 4.1. Nrf2 and Bmal1 and Klf9 transcription factors in Prdx6 regulation

Bmal1, a core circadian clock component, has recently emerged as a key regulator of antioxidant genes, including Prdx6 (Chhunchha et al., 2020; Pekovic-Vaughan et al., 2014). Bmal1 modulates responses to oxidative stress via interaction with E-box elements in gene promoters and is present in the promoters of the Prdx6 and Nrf2 genes (Chhunchha et al., 2020; Early et al., 2018; Pekovic-Vaughan et al., 2014). Chromatin immunoprecipitation assays showed Bmal1 binding to E-box sequences in the Prdx6 gene promoter, functioning synergistically with Nrf2 to increase Prdx6 transcription (Chhunchha et al., 2022a). Notably, Bmal1/Nrf2/Prdx6 and other Phase II antioxidants show rhythmic expression in vivo in mouse lenses, with expression patterns inversely correlated with ROS levels (Chhunchha et al., 2022a). This dual regulatory mechanism strengthens cellular defenses. Studies have shown that the depletion of either brain and muscle aryl hydrocarbon receptor nuclear translocator-like protein 1 (Bmal1) or Nrf2 significantly reduces Prdx6 expression, underscoring their synergistic role (Fig. 4). Importantly, Klf9, which is also known as basic transcription element binding protein 1 (BTEB1), has been found to act as a negative regulator of antioxidant genes such as *Prdx6* (Bagati et al., 2019; Bonett et al., 2009; Chhunchha et al., 2022a; McConnell et al., 2007). Under oxidative stress, Klf9 is upregulated via Nrf2 and suppresses the expression of genes such as *Prdx6*, thereby aggravating oxidative damage (Chhunchha et al., 2019, 2022a, 2022b; Imataka et al., 1992; Zucker et al., 2014). Inhibition of Klf9 restores Prdx6 expression in stressed cells, thereby improving antioxidant defenses (Figs. 1 and 4) (Chhunchha et al., 2019, 2022a).

### 4.2. Lens epithelium-derived growth factor (LEDGF)

LEDGF is a stress-inducible transcriptional protein that plays a vital role in maintaining cellular homeostasis and protecting cells against oxidative stress (Basu et al., 2012; Fatma et al., 2001; Shinohara et al., 2002; Singh et al., 1999, 2001). It regulates various stress-related genes, including antioxidant proteins such as Prdx6. LEDGF activates Prdx6 transcription by binding to heat shock elements (HSEs) and stress-related elements (STREs) within its promoter region, thereby enhancing cellular resistance to oxidative stress (Fatma et al., 2001; Singh et al., 2001). LEDGF is involved in diverse cellular functions, and its activity is regulated by dynamic SUMOylation at lysine 364. SUMOylation suppresses LEDGF's DNA binding and transcription activity, whereas deSUMOylation or mutation at lysine 364 enhances its transcription, increases small heat shock protein levels, and promotes cell survival. SUMOylation of LEDGF serves as an important post-translational regulatory mechanism that modulates its transcriptional activity (Ishihara et al., 2012). LEDGF expression is epigenetically regulated (Bhargavan et al., 2024) via methylation of CpG islands in its promoter, particularly near specificity protein 1 (Sp1) binding sites. This methylation represses LEDGF expression during aging and in response to oxidative stress. Loss of LEDGF correlates with increased expression of DNA (Cytosine-5)-Methyltransferase (DNMT)1, DNMT3a, DNMT3b, and methyl-CpG-binding protein (MeCP2), which promote methylation and silencing of the LEDGF gene. This epigenetic regulation of LEDGF directly impacts Prdx6 expression and the ability of cells to counteract oxidative damage (Bhargavan et al., 2024).

### 4.3. Specificity protein 1 (Sp1)

Sp1 is a critical transcription factor that regulates Prdx6 expression by binding to GC-rich promoter regions under normal conditions (Chhunchha et al., 2018). Sp1 expression decreases significantly in aging (Singh et al., 2012) and oxidative stress conditions (Chhunchha et al., 2011, 2014; Singh et al., 2012; Spengler and Brattain, 2006). Aberrant SUMOylation of Sp1 during oxidative stress and aging impairs its ability to promote Prdx6 expression (Chhunchha et al., 2018). Notably, a SUMOylation-deficient Prdx6 mutant exhibited enhanced enzymatic activity and stability, circumventing SUMO-mediated repression of Sp1-dependent Prdx6 expression in aged LECs (Chhunchha et al., 2018). This deficiency facilitates the retention of protective functions by Prdx6, mitigating oxidative damage in aging cells (Chhunchha et al., 2018).

## 5. Role of Prdx6 in cell signaling in lens

Prdx6 functions as an antioxidant enzyme and as a key regulator of multiple cells signaling pathways involved in oxidative stress, such as inflammation, apoptosis, ferroptosis, and tissue repair. Below, we present the critical signaling mechanisms modulated by Prdx6.

### 5.1. TGF-β signaling and ROS signaling

Prdx6 regulates TGF-β signaling, which is crucial for cell growth, differentiation, and tissue repair (Fatma et al., 2005; Kubo et al., 2006, 2010). In the lens, elevated TGF-β signaling is linked to cataract development, particularly under oxidative stress (Fatma et al., 2005, 2009b; Lovicu et al., 2002; McAvoy et al., 2000). Prdx6 transcription is finely regulated through interactions with transcription factors such as mothers against decapentaplegic homolog (Smad)3 and NF-*κ*B (Fatma et al., 2009b), which can either repress or promote its expression in response to oxidative and TGF-β signaling. In Prdx6 regulation, Smad3 binds to two repressive Smad-binding elements (SBEs) within the Prdx6 promoter, suppressing its transcription (Fatma et al., 2009b). In *Prdx6*^*−/−*^ LECs, nuclear accumulation of phosphorylated Smad3 (pSmad3) causes this repression. Notably, neutralization of TGF-β1 restored Prdx6 promoter activity and expression. This repression is especially relevant in age-related diseases, such as cataracts and fibrosis, where increased TGF-β signaling coincides with diminished antioxidant defenses, such as Prdx6 (Fatma et al., 2009a, 2009b; Hu et al., 2018).

### 5.2. Nucleotide oligomerization domain (NOD)-like receptor family pyrin domain containing 3 (NLRP3) inflammasome and ROS signaling

The NLRP3 inflammasome, a prominent member of the NOD-like receptor family, plays a critical role in initiating inflammatory signaling associated with aging and age-related diseases (Rea et al., 2018). ROS overproduction triggers NLRP3 inflammasome activation, promoting the assembly of NLRP3, ASC, and caspase-1 complexes that induce cytokine activation (Dominic et al., 2022; Kanneganti et al., 2006; Rea et al., 2018). Prdx6 antioxidant activity neutralizes ROS and prevents aberrant NLRP3 activation. In *Prdx6*^*−/−*^ LECs, ROS accumulation activates NLRP3 inflammasome, causing pyroptosis, which is exacerbated by aging, with Prdx6 deficiency leading to oxidative stress-driven NLRP3 activation and an inflammatory cascade (Chhunchha et al., 2023b). Notably, overexpression of Klf9 elevates NLRP3 at mRNA and protein levels, acting as a pro-oxidant that amplifies the inflammasome response. Conversely, Klf9 knockdown normalized ROS levels and reduced NLRP3 activation, thereby protecting cells from oxidative damage. In addition, in *Prdx6*^*−/−*^ LECs, elevated ROS levels induce ER stress, which further triggers Nlrp3 inflammasome activation (Chhunchha et al., 2023b).

## 6. Prdx6 expression in the lens and therapeutic implications

The eye is constantly exposed to ROS-driven oxidative stress, which is generated by various internal and external factors. If ROS are not adequately neutralized by antioxidant systems, excessive oxidative stress leads to pathological signaling that drives age-related lens dysfunction and cataract formation. Despite extensive research and multiple clinical trials, enzymatic and non-enzymatic antioxidant therapies have, without exception, failed to cure or halt cataract progression (Al-Madhagi and Masoud, 2024; Davies and Holt, 2018; Steinhubl, 2008). Several factors likely underlie these failures, including the suboptimal selection of antioxidant compounds based on availability rather than efficacy, inadequate treatment duration, and the complex and multifactorial nature of oxidative injury in the lens. Importantly, pharmacokinetic limitations represent a major obstacle, as numerous studies have indicated that exogenously administered antioxidants achieve substantially lower concentrations in the lens than in other ocular compartments, such as the aqueous humor or vitreous body, owing to the avascular nature of the lens and diffusion barriers that restrict drug penetration (Beebe et al., 2014; Lou, 2003; Spector, 1995). Consequently, many antioxidant compounds fail to reach concentrations sufficient to effectively quench ROS within lens epithelial cells (Davies and Holt, 2018; Steinhubl, 2008). For example, vitamin E, one of the most widely studied antioxidants, has shown limited or no efficacy in preventing cataracts in clinical and epidemiological studies, likely due to insufficient intra-lenticular availability, instability at the target site, and a mismatch between its antioxidant mechanism and the predominant oxidative injury in the aging lens (Sideri et al., 2019; Spector, 2000). These limitations highlight the need to better understand the specific mechanisms of oxidative damage in the lens and to design therapeutic strategies that ensure adequate target engagement in the lens. Notably, the lens and LECs are intrinsically equipped with robust endogenous antioxidant defense systems, including Prdx6; however, these systems progressively deteriorate with aging, thereby increasing susceptibility to oxidative damage and cataractogenesis. Based on this framework, we propose that effective therapeutic strategies should focus on restoring or augmenting endogenous antioxidant capacity, rather than relying solely on exogenous ROS scavengers. Accordingly, therapeutic strategies aimed at restoring lenticular redox homeostasis should focus on enhancing endogenous antioxidant capacity, either by increasing Prdx6 availability through targeted delivery approaches or pharmacologically inducing Prdx6 expression using agents such as metformin or hydralazine. In this context, the combined modulation of complementary protective pathways may further improve therapeutic efficacy. Among these strategies, we emphasize “transcription inductive therapy” aimed at reactivating endogenous lenticular Prdx6 using Food and Drug Administration (FDA)-approved drugs or their mimetics. Experimental studies have demonstrated that such agents can reach the lens following topical administration in animal models and effectively activate the Nrf2/ARE–Prdx6 pathway, leading to attenuation of oxidative injury and preservation of lens transparency (Chhunchha et al., 2023a; Kubo et al., 2017; Singh et al., 2023). In contrast, the direct delivery of proteins or nucleic acids faces significant challenges related to bioavailability, stability, half-life, and patient acceptability, particularly given the invasive nature of intraocular injections. Collectively, these findings support the concept that pharmacological reactivation of endogenous antioxidant defenses, rather than supplementation with exogenous antioxidants, is a more viable and physiologically relevant strategy for mitigating oxidative stress-driven lens pathology. Such approaches may not only offer therapeutic potential but also serve as powerful tools for dissecting the molecular pathways underlying age-related cataract formation. From a translational perspective, several challenges must be addressed before Prdx6-centered strategies can be clinically implemented in humans. At present, no bona fide small-molecule Prdx6 agonists have been established, largely because Prdx6 activity is regulated by complex post-translational modifications and redox-dependent conformational states rather than by a single, well-defined ligand--binding pocket. Direct administration of recombinant Prdx6 protein or nucleic acid–based approaches, such as Prdx6 mRNA or gene delivery, are theoretically feasible but face substantial practical limitations. These include poor intra-lenticular penetration, limited stability and half-life, potential immunogenicity, and the need for invasive intraocular delivery of the drug. In addition, the durability of expression, safety considerations, and patient acceptability represent significant barriers, particularly in the context of a non–life-threatening condition, such as cataracts. In contrast, pharmacological induction of endogenous Prdx6 expression is a more practical and physiologically aligned strategy. By leveraging intact cellular regulatory networks, this approach avoids many bioavailability, safety, and delivery constraints associated with protein- or nucleic acid–based therapeutics. Accordingly, modulation of upstream regulatory pathways controlling Prdx6 expression may offer a more feasible route for translational exploration in age-related lens pathology.

### 6.1. Comparative assessment of Prdx6-targeted therapeutic strategies

Current strategies to enhance Prdx6-dependent antioxidant defense differ substantially in their mechanisms of action, translational readiness, and associated challenges. Pharmacological inducers such as metformin, hydralazine (Hyd), and selected natural compounds offer the advantage of established safety profiles and non-invasive administration, positioning them closest to clinical translation, although their effects on Prdx6 are indirect and context-dependent (Chhunchha et al., 2019, 2022b, 2023a; Kubo et al., 2017). In contrast, protein-based approaches, including protein transduction domain (PTD)–mediated delivery of Prdx6 (e.g., transcriptional activator of transcription (TAT) -based strategies) and nanoparticle-assisted formulations, provide direct proof-of-concept that restoration of Prdx6 activity can rescue oxidative stress–driven lens pathology (Fatma et al., 2008; Kubo et al., 2008). However, these strategies remain largely preclinical and are limited by their delivery efficiency, stability, potential immunogenicity, and regulatory considerations. Thus, while protein- and nanotechnology-based approaches are invaluable for mechanistic validation and technology development, the pharmacological induction of endogenous Prdx6 currently represents the most practical avenue for near-term translational exploration.

## 7. Induction of naturally occurring endogenous Prdx6 by inducers

### 7.1. Metformin

The antidiabetic drug metformin delays aging, inhibits age-related pathological changes, and reduces oxidative stress (Araujo et al., 2017; Matsiukevich et al., 2017). Lenses/LECs are enriched with Prdx6, the expression of which declines with advancing age, as observed in rats, mice, and humans (Fatma et al., 2005; Hasanova et al., 2009; Kubo et al., 2006). Prdx6 levels are also decreased in diabetic rat lenses and human LECs when treated with high glucose, and these cells showed excessive levels of ROS (Kubo et al., 2004, 2005), suggesting that Prdx6 level reduction may contribute to cataract formation. Metformin is known to exert its protective effect via upregulating the antioxidant pathway. Metformin enhances the nuclear translocation of Nrf2 and its binding to AREs within the Prdx6 promoter (Chhunchha et al., 2022b), significantly upregulating Prdx6 transcription, particularly in cells under oxidative stress (Chhunchha et al., 2022b). Experiments using Nrf2-deficient cells have confirmed that Nrf2 is essential for metformin-induced Prdx6 expression (Chhunchha et al., 2022b), as these cells showed drastically reduced Prdx6 transcription despite metformin treatment. Therefore, the Nrf2-Prdx6 pathway is critical in tissues such as the lens, where oxidative stress contributes to cataract formation. Moreover, metformin increases Bmal1 binding to E-box elements in the Prdx6 promoter, enhancing its transcription (Chhunchha et al., 2022b). Bmal1-Nrf2 cooperation is vital for peak activation of the Prdx6 promoter, as *Bmal1*-deficient cells exhibit markedly reduced Prdx6 expression, even with metformin treatment. In aging LECs, where oxidative stress is elevated and Bmal1 activity declines, metformin restores Bmal1 and Nrf2 functions. This restoration is crucial for protecting lens cells from age-related oxidative damage, which drives cataractogenesis. In addition to Prdx6, metformin upregulates other antioxidant genes regulated by Nrf2 and Bmal1, including catalase, superoxide dismutase, and GPx (Chhunchha et al., 2022b). Metformin's ability to restore antioxidant defenses via the Nrf2-Bmal1-Prdx6 axis may offer a novel approach to mitigating aging and oxidative damage (Figs. 1 and 4).

### 7.2. Hydralazine (Hyd)

Hyd, an FDA-approved antihypertensive drug, has recently been identified as a potent activator of the Nrf2/ARE pathway (Chang et al., 2022; Dehghan et al., 2017). Hyd treatment promotes the nuclear translocation of Nrf2 and enhances its binding to AREs, resulting in the upregulation of antioxidant genes, including Prdx6, in LECs. Activation of the Nrf2/ARE pathway by Hyd protects LECs from oxidative stress-induced damage, thereby preventing or delaying cataract formation. Moreover, Hyd treatment increases Prdx6 transcription and enzymatic activity (Chhunchha et al., 2023a), which helps maintain cellular homeostasis in LECs, prevents oxidative damage, and supports lens transparency (Figs. 1 and 4).

### 7.3. Natural agents and Prdx6 expression

#### 7.3.1. Curcumin

Curcumin, renowned for its antioxidant properties, enhances the activity of the transcription factor Sp1 (Vallianou et al., 2015), leading to increased Prdx6 transcription (Bu et al., 2023; Chhunchha et al., 2011; Jia et al., 2017). Curcumin promotes Sp1 binding to specific sites within the Prdx6 promoter, thereby upregulating Prdx6 expression in LECs under oxidative stress (Chhunchha et al., 2011). This mechanism highlights curcumin's role in boosting Prdx6 as part of its antioxidant defense, which is particularly important in the lens for preventing cataract formation by attenuating oxidative damage (Figs. 1 and 4).

#### 7.3.2. Ginkgolic acid (GA)

GA, a natural compound derived from *Ginkgo biloba* leaves, modulates redox signaling and protects against oxidative stress-induced cellular damage by inhibiting protein SUMOylation (Brackett et al., 2020; Chhunchha et al., 2018; Fukuda et al., 2009; Liu et al., 2020). It rescues Prdx6 from aberrant SUMOylation, restoring its antioxidant activity and protecting LECs from oxidative damage (Chhunchha et al., 2018). GA upregulated Prdx6, further strengthening the antioxidant defense of LECs. Beyond restoring Prdx6 activity, GA activates the transcription factor Sp1, an activator of Prdx6 transcription, by reversing its SUMOylation (Chhunchha et al., 2018). This dual action on both Prdx6 and Sp1 underscores its therapeutic potential in preventing cataracts by preserving lens homeostasis (Figs. 1 and 4).

#### 7.3.3. Sulforaphane (SFN)

SFN is a naturally occurring isothiocyanate found in cruciferous vegetables such as broccoli, Brussels sprouts, and kale. It is widely recognized for its potent antioxidant, anti-inflammatory, and anticancer properties (Zanichelli et al., 2012). SFN activates the Nrf2-antioxidant protective pathway (Hanlon et al., 2008; Houghton et al., 2016). In LECs, SFN treatment significantly upregulated Nrf2-Prdx6, correlating with increased resistance to H_2_O_2_-induced oxidative stress (Kubo et al., 2017). To achieve this, SFN stabilizes Nrf2 in the nucleus, facilitating its binding to AREs in the Prdx6 promoter (Kubo et al., 2017). Pretreatment of mouse fibroblasts and LECs with non-toxic SFN doses (3–6 μM) increased their resistance to oxidative stress induced by paraquat, H_2_O_2_, or ultraviolet B in an Nrf2-dependent manner (Higgins et al., 2009; Kubo et al., 2017). However, higher SFN doses (>6 μM) paradoxically activated death signaling via overactivation of the Nrf2/ARE-mediated transcription factor Klf9, which represses Prdx6 expression and increases ROS load and cell death (Chhunchha et al., 2019). These findings reveal a dose-dependent dual role of SFN in modulating the Nrf2/Klf9/Prdx6 axis in LECs. In this scenario, SFN-mediated toxicity can be controlled by subsiding the abnormal activity and effects via combination therapies such as SFN plus genetic or pharmacological suppressors of Klf9. Based on our studies (Chhunchha et al., 2019, 2022a), we propose that for therapeutic interventions in aging and oxidative disorders, combining Klf9 knockdown (e.g., Klf9-ShRNA) with Nrf2 inducers such as SFN may represent a promising strategy to maintain lens biology/transparency (Figs. 1 and 4). Moreover, several studies have demonstrated that SFN is a potent cytoprotective with diversified functions, and its ability depends on its concentration and the cellular background (Ferreira de Oliveira et al., 2014; Houghton et al., 2016; Zanichelli et al., 2012; Zucker et al., 2014). Thus, as SFN possesses cell-specific activity (from cell survival to cell death), a toxicity assay should be conducted for its activity in specific cell types before its delivery to specific organs/tissues. Further, SFN's lipophilic nature and low molecular weight readily enable passive diffusion into cells/tissue and are rapidly absorbed and bioavailable for its effect. However, SFN delivery to the eye in the form of eye drops may not be feasible, as SFN may not reach the lenses at an optimum concentration to exert its protective effect. From a drug development perspective, these findings highlight the narrow therapeutic window of SFN and underscore the importance of precise dose optimization to maximize protective effects while avoiding toxicity. Strategies that improve target specificity or enable controlled, low-level activation of the Nrf2–Prdx6 axis, rather than global Nrf2 overactivation, may be essential to ensure safety while improving bioavailability in future therapeutic applications. Thus, SFN may not be a suitable phytochemical to delay or prevent oxidative stress-induced lens pathology.

## 8. Therapeutic potential of Prdx6: direct delivery to the eye

The crystalline lens is an avascular organ, relying on diffusion from the aqueous humor for nutrient supply and waste removal. This unique structure poses a major challenge for drug delivery, as many therapeutic agents cannot effectively penetrate the dense lens tissue. Conventional delivery methods, including oral administration and eye drops, have limited success in delivering therapeutic proteins to the lens at sufficient levels. Therefore, innovative delivery systems are essential to effectively target LECs with antioxidant proteins such as Prdx6, aiming to prevent oxidative stress and the resulting cataract formation.

### 8.1. Protein transduction domain (PTD)-linked Prdx6: A novel approach to protein delivery

One promising strategy to overcome the challenge of delivering therapeutic proteins to the lens involves the use of TAT protein from human immunodeficiency virus (HIV). The TAT protein contains an 11-amino-acid PTD (GRKKRRQRRR) that facilitates the concentration-dependent transport of large molecules across cell membranes (Becker-Hapak et al., 2001; Kubo et al., 2008, 2009). The TAT PTD has been widely employed to deliver various therapeutic proteins both in vitro and in vivo, and its application to lens proteins such as Prdx6 marks a significant advance in cataract prevention (Becker-Hapak et al., 2001; Chhunchha et al., 2018, 2021, 2022a, 2023b; Kubo et al., 2008, 2009). Fusion of Prdx6 with TAT-PTD (TAT-hemagglutinin epitope tag (HA)-Prdx6) allows the chimeric protein to cross cell membranes and localize within LECs. In vitro studies have demonstrated that TAT-HA-Prdx6 effectively reduces ROS and prevents apoptosis in LECs exposed to oxidative stress (Chhunchha et al., 2018, 2021, 2022a, 2023b) (Figs. 1 and 4). In animal models, TAT-HA-Prdx6 administered via subconjunctival or anterior chamber injections in rats and rabbits successfully penetrated the lens, confirming its ability to overcome lens tissue barriers and reach LECs. Notably, subconjunctival administration of TAT-HA-Prdx6 in spontaneously cataractous rats prior to cataract onset delayed cataract progression, indicating effective in vivo protection against oxidative stress (Chhunchha et al., 2021; Kubo et al., 2008). Although TAT-PTD–Prdx6 delivery is unlikely to represent a near-term clinical therapy due to its invasive nature and protein-based limitations, these studies provide critical proof-of-concept evidence demonstrating that restoration of Prdx6 activity is sufficient to rescue oxidative stress–driven lens pathology.

### 8.2. SUMOylation-deficient Prdx6 is an excellent therapeutic agent

The discovery that SUMOylation-deficient Prdx6 exhibits enhanced protective function has important implications for therapeutic interventions aimed at combating oxidative stress-related diseases (Chhunchha et al., 2017, 2018, 2021). In vitro studies using recombinant Prdx6 mutants with K122R and K142R substitutions have demonstrated improved cellular delivery and increased protection against oxidative stress (Chhunchha et al., 2017, 2018). Furthermore, TAT-HA-Prdx6 analog-loaded SUMOylation-deficient poly lactic-co-glycolic acid (PLGA) nanoparticles (NPs) were cytocompatible with human, mouse, and Shumiya cataract rat (SCR) LECs and lenses (Chhunchha et al., 2021). Delivery of these modified Prdx6-NPs delayed lens opacity in SCR models more effectively than wild-type Prdx6 (Chhunchha et al., 2021). Specifically, TAT-HA-Prdx6-NPs achieved a 35 % greater reduction in oxidative stress compared with wild-type Prdx6-NPs, underscoring the advantage of nanoparticle-mediated delivery in enhancing Prdx6 bioavailability and efficacy (Chhunchha et al., 2021). This localized, non-invasive delivery system holds promise for maintaining redox homeostasis in the lens, protecting LECs from oxidative damage, and potentially delaying or preventing cataract formation (Figs. 1 and 4). Accordingly, SUMOylation-deficient and nanoparticle-formulated Prdx6 should be viewed primarily as advanced experimental platforms that inform future delivery strategies, rather than as immediately translatable clinical interventions.

## 9. Conclusions

The significant roles of Prdx6 have gained increasing recognition as Prdx6 is a crucial defender against oxidative stress in the lens, functioning both to scavenge ROS and repair damaged cell membranes. However, its protective capacity declines with age due to PTMs and reduced expression, increasing susceptibility to cataract formation. Insights into Prdx6 regulation via transcription factors such as Nrf2 and Sp1 provide key avenues for mitigating oxidative damage in aging lenses. Innovative therapeutic strategies aimed at enhancing Prdx6 activity show great promise for cataract prevention (Figs. 1-4). Agents such as metformin, Hyd, sulforaphane, GA, and curcumin help to prevent detrimental PTMs, amplifying Prdx6's protective effects. Moreover, novel delivery methods—including TAT-mediated protein transduction, nanoparticle-based systems, and SUMOylation-deficient Prdx6 mutants—effectively overcome biological barriers to target LECs.

Ongoing exploration of Prdx6 as a therapeutic target has the potential to revolutionize antioxidant treatments for age-related cataracts. By combining molecular regulators with advanced delivery technologies, future therapies may halt cataract progression and restore lens transparency, significantly enhancing quality of life for millions affected by this condition.

## Glossary

PRDX6: peroxiredoxin 6
ROS: reactive oxygen species
GPX: glutathione peroxidase
PTMs: post-translational modifications
LECs: lens epithelial cells
ARDs: age-related diseases
AOP2: antioxidant protein 2
Nrf2: nuclear factor erythroid 2-related factor 2
SUMO: Small Ubiquitin-like Modifier
SUMO conjugation: SUMOylation
H_2_O_2_: hydrogen peroxide
Cys: Cysteine
Trx: thioredoxin
GSH: glutathione
aiPLA_2_: Ca^2+^-independent PLA_2_
LPCAT: lysophosphatidylcholine acyl transferase
PLOOHs: phospholipid hydroperoxides
GST: glutathione S-transferase
PLA2: phospholipase A_2_
ER: endoplasmic reticulum
UPR: unfolded protein response
Prdx6 −/−: Prdx6 deficiency/ Prdx6 knockout
TGF-β2: transforming growth factor-β2
EMT: epithelial-mesenchymal transition
ARC: age-related cataract
nanoUPLC-ESI-q-TOF MS/MS: nano–ultra-performance liquid chromatography-–electrospray ionization–quadrupole time-of-flight tandem mass spectrometry (nanoUPLC-ESI-q-TOF MS/MS)
Modi: Modification Identification
MODmap: Modification Mapping
Prdx6WT: wild-type Prdx6
T177: threonine 177
Cys47: catalytic cysteine residue
HIF: hypoxia-inducible factor
NF-κB: nuclear factor-κB
Ap1: activator protein 1
c-Myc: c-myelocytomatosis oncogene
Klf9: Krüppel-like factor 9
Keap1: kelch-like ECH-associated protein 1
ARE: antioxidant response element
Bmal1: brain and muscle aryl hydrocarbon receptor nuclear translocator-like protein 1
BTEB1: basic transcription element binding protein 1
LEDGF: lens epithelium-derived growth factor
HSEs: heat shock elements
STREs: stress-related elements
Sp1: specificity protein 1
DNMT: DNA (Cytosine-5)-Methyltransferase
MeCP: methyl-CpG-binding protein
Smad: mothers against decapentaplegic homolog
SBEs: Smad-binding elements
NOD: nucleotide oligomerization domain
NLRP3: NOD-like receptor family pyrin domain containing 3
FDA: Food and Drug Administration
Hyd: hydralazine
PTD: protein transduction domain
TAT: transcriptional activator of transcription
GA: Ginkgolic acid
SFN: sulforaphane
HIV: human immunodeficiency virus
HA: hemagglutinin epitope tag
PLGA: poly lactic-co-glycolic acid
NPs: nanoparticles
SCR: Shumiya cataract rat
